# MicroRNAs: where brilliance, perseverance, and ambition converged

**DOI:** 10.1172/JCI189625

**Published:** 2024-12-10

**Authors:** Rares Drula, George A. Calin

**Affiliations:** 1 Translational Molecular Pathology Department,; 2 Leukemia Department, and; 3 Center for RNA Interference and Non-Coding RNAs, The University of Texas MD Anderson Cancer Center, Houston, Texas, USA.

Victor Ambros at the University of Massachusetts Medical School and Gary Ruvkun at Harvard University were awarded the 2024 Nobel Prize in Medicine and Physiology for the discovery of microRNAs. This “groundbreaking discovery revealed a completely new principle of gene regulation that turned out to be essential for multicellular organisms, including humans” (1). This is a well-deserved and highly anticipated recognition for a seminal discovery published 31 years ago in back-to-back *Cell* publications (2, 3). This landmark finding changed the way we understand gene expression regulation among most multicellular organisms (4) and had a huge impact in deciphering the mechanisms of many diseases.

## Into the unknown — cracking the interaction of lin-4 and lin-14

The story of lin-4 began in the mid-1970s in Sydney Brenner’s lab at Cambridge, where the gene was discovered in *Caenorhabditis elegans*. Lin-4 was particularly interesting owing to the observation that animals bearing a loss-of-function mutation (e912) disrupted the timing of developmental transition. By this point, lin-4 was recognized as a “heterochronic” gene — a master regulator of sequence of cell fate decisions and timing (5). Robert Horvitz, observing similar effects, independently published a detailed account of the developmental defects caused by lin-4 mutations. Victor Ambros, then a postdoctoral fellow in Horvitz’s lab, identified lin-14 as another heterochronic gene. Notably, he observed that null alleles of lin-14 produced developmental defects opposite to those seen in lin-4 mutants, suggesting that lin-4 likely acted as a negative regulator of lin-14. At the time, the prevailing genomic paradigm held that both lin-4 and lin-14 encoded proteins, and Gary Ruvkun, working under the joint mentorship of Walter Gilbert and Horvitz, along with Ambros, embarked on the journey to clone and characterize the products of these genes (6).

**Figure 1:**
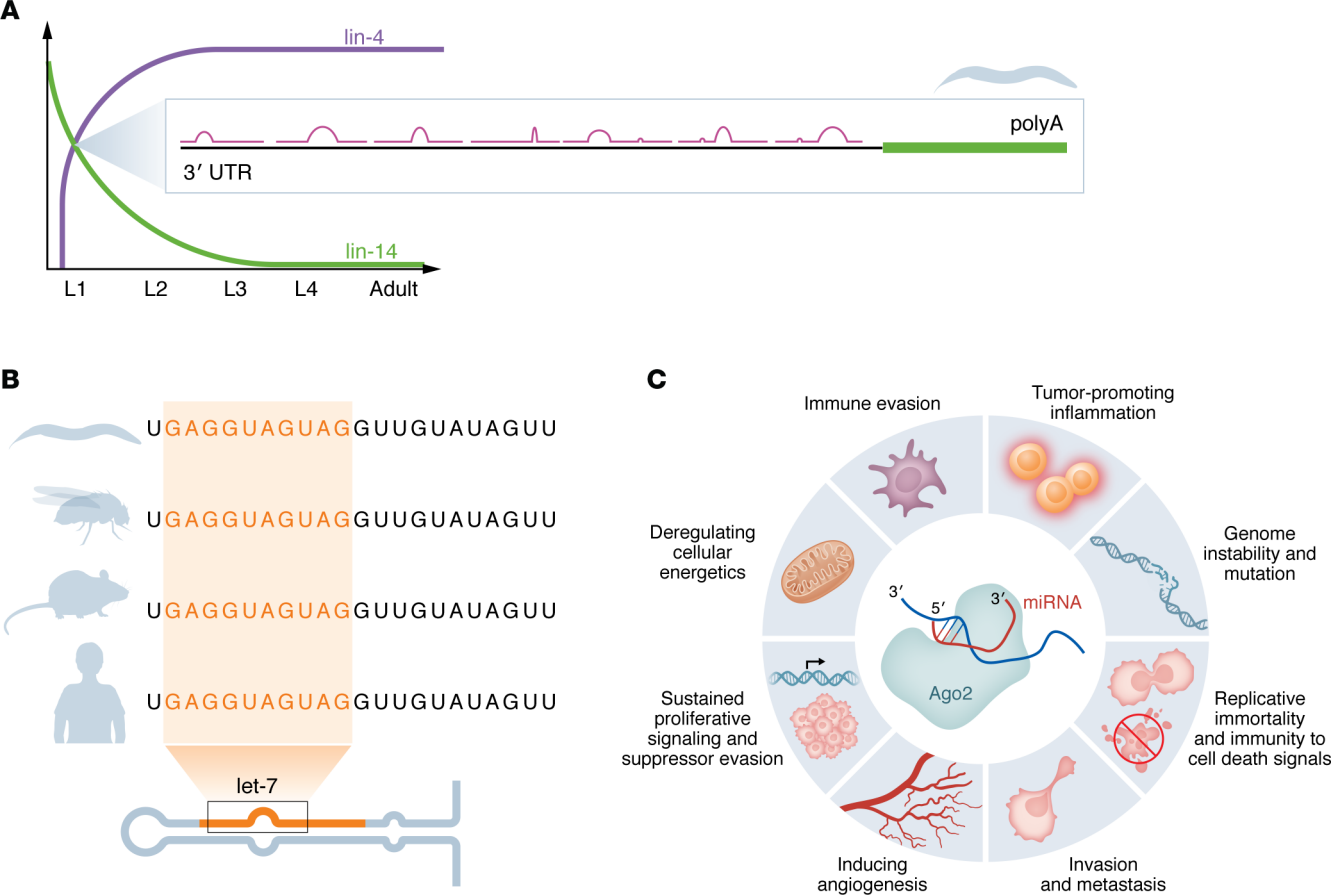
microRNA research: from sequences to hallmarks. (**A**) The regulatory relationship between lin-4 and lin-14, as defined by the discoveries of Ambros and Ruvkun, highlighting the seven complementary binding sites of lin-4 on the 3′ UTR of lin-14. (**B**) The evolutionary conservation of let-7 sequences is displayed across key model organisms, including *Caenorhabditis elegans*, *Drosophila melanogaster*, mice (*Mus musculus*), and humans (*Homo sapiens*). (**C**) MicroRNAs at the intersection of cancer hallmarks, highlighting their involvement in processes such as immune evasion, angiogenesis, metastasis, and cellular proliferation. While significant progress has been made in understanding their roles, many aspects remain undefined, emphasizing the need for further research into their complex contributions to tumor biology and cancer progression.

Slowly, a regulatory model started to be defined, but there were major caveats regarding both the temporality and nature of the biological interactions. Both Ambros and Ruvkun, who at this point were embarking on independent careers at Harvard Medical School and at Massachusetts General Hospital, respectively, envisioned a protein that might engage the *lin-14* 3′ untranslated region (UTR), as Ruvkun previously noted that proteins could be translated from the mutants in 3′UTR and retained long after their corresponding larval stage, clearly indicating a posttranscriptional mechanism. Ambros and Rosalind Lee were puzzled by the fact that inducing frameshift mutations in the lin-4 sequence had no functional effects, particularly given the gene’s exceptionally short open reading frame. This evidence strongly supported that lin-4 did not encode a translatable mRNA but instead produced two small transcripts, 22 and 61 nucleotides in length (2). At the time, as the term “microRNA” had not yet been coined, they were referred to as small regulatory RNAs. The breakthrough moment occurred on June 11, 1992, when Ambros and Ruvkun exchanged the lin-4 and lin-14 3′ UTR sequences and immediately noticed the antisense complementarity between them, each reading the complementary sequences to the other over the phone, practically in unison. “That was a very happy shared moment,” as said by Ambros remembering of this unforgettable moment (5). Years of work culminated in clarity about the mechanism of regulation — a breakthrough that Ruvkun later described as a “classic eureka moment” (7) (Figure 1A). What may now seem like a straightforward discovery was, in reality, the result of two decades of intense effort and unwavering passion of all the contributing minds, including Bruce Wightman and Ilho Ha from Ruvkun’s lab, Rosalind Lee and Rhonda Feinbaum from Ambros’s team, and collaborators from the *C*. *elegans* field, such as Prema Arasu, Joe Gatto, John Giusto, Thomas Bürglin, and Phil Olsen.

## The stage was set — waiting for an RNA breakthrough

These small regulatory RNAs remained largely overlooked after their initial discovery in *C*. *elegans*, as the lack of genomic and regulatory context in higher eukaryotes deeming them no more than atavistic regulatory transcripts. The reality was that several defining findings were already setting the stage for the incoming dogmatic shift of RNAs significance, from bystanders in genetic communication between DNA to proteins to master regulators of protein expression. Of note, Carl Woese proposed “the RNA World Hypothesis” in 1967, a concept expanded further by the same Walter Gilbert (8) who trained Ruvkun. The existence of RNA transcripts without a protein coding function has been known since the 1980s, where, in prokaryotes, the regulation of gene expression by noncoding antisense RNAs was well studied (9), along with the description of ribozymes and RNA molecules with catalytic activity from a ciliated protozoan (10) and from bacteria (the RNA subunit of ribonuclease P) (11).

By that time, genomic alterations were already defined as hallmarks of various diseases, particularly cancer, making a future connection with microRNAs possible. For example, a lesser-known 1989 *Proceedings of the National Academy of Sciences of the United States of America* publication — four years before the official discovery of microRNAs — reported a case of aggressive prolymphocytic leukemia in a patient with an abnormal karyotype involving a t(14;18) translocation (12). This rearrangement resulted in a truncated *MYC* gene that was highly regulated by BCL3, a presumed regulatory gene driving aggressive B cell leukemia. The protein encoded by “that” BCL3 gene was never cloned. Only years later, the precursor of microRNA-142, a frequently overexpressed microRNA in B cell lymphomas and leukemias, was mapped to the “BCL3” gene (13).

Yet, it wasn’t until the early 2000s when Ruvkun’s group discovered let-7, another *C*. *elegans* heterochronic RNA, with remarkably conservation in a wide range of species, including humans (14). This sparked the huge interest in the microRNA study (Figure 1B) shortly after; the Bartel, Tushl, and Ambros groups reported simultaneously the cloning of multiple microRNAs from worms and mammals (15–17), proving that microRNAs regulate gene expression across many species. Finally, the convergence with the discovery of small interfering RNAs and RNA interference in late 90s (18, 19) ushered in a new age in molecular biology.

Although all these findings were available for the research community, the discovery of genetic alterations associated with microRNAs in human cancers came by surprise. These were reported about a decade after the finding of microRNAs and consisted of homozygous and heterozygous deletions of miR-15a and miR-16 from chromosome 13 in the most frequent leukemia, the chronic lymphocytic leukemia (20). This was the ultimate proof of the microRNAs true involvement in human pathology, event that would define this research area to this day.

## The great microRNA rush

The impact of Ambros and Ruvkun’s discovery was profound, sparking a surge of interest in microRNAs that rapidly extended beyond the niche of developmental biologists. Defining the microRNA processing pathway and the importance of the seed sequence for assessing microRNA function allowed the creation of many in silico tools for mRNA target prediction. These tools allowed microRNA research to gain widespread attention (21), with more than 175,000 manuscripts on the topic of microRNAs and over 80,000 on the topic of microRNAs and cancer being published in the last two decades. While it is beyond the scope of this Viewpoint to catalog all the major discoveries of this period, key findings have firmly established the involvement of microRNAs in processes such as metastasis, hypoxia, neurodevelopment, and immune function as well as their associations with critical gene expression regulators like TP53 (22). These discoveries underscore their role in virtually all hallmarks of cancer (Figure 1C). Furthermore, the detection of microRNAs within extracellular vesicles has been recognized as pivotal, highlighting their function as mediators of intercellular communication and expanding their function outside of the cells (23).

Conversely, the seemingly straightforward experimental design, combined with the widespread availability of in vitro tumor models, facilitated a surge in functional studies adhering to the familiar framework of “miR-*x* targets gene *y* in disease *z*.” In many cases, a simple correlation between a specific microRNA and a target gene was deemed sufficient to establish functional relevance. However, most interpretations relied heavily on superficial parallels and did not account for multiple target genes, often resulting in conclusions that lacked depth and robustness.

The publication surge contributed to a wealth of mechanistic data that generated an inflated view of microRNAs as important therapeutic targets. However, as the first clinical trials began, the limitations of these early findings became evident. While preclinical studies in microRNA therapeutics have shown promise, with several advancing to clinical trials, none have managed to reach phase III or gain FDA approval to this day. The failure of the first microRNA-based therapeutic, MRX34 — a liposomal formulation of miR-34a intended for the treatment of solid tumors and purportedly targeting over 30 oncogenes — served as a wake-up call for the emerging field. The trial, halted in 2016 due to severe immune-related toxicities, highlighted the challenges of microRNA-based therapies. On the other hand, multiple RNAi and mRNA-based therapies have gained FDA approval (24), surging again the hope of successful microRNA-based therapeutics.

Certainly, microRNA-based therapeutics have lagged behind other RNA-based therapeutics exactly owing to their inherent biological functions. A single microRNA never regulates a single gene; thus the framework for microRNA-based therapies must account for complex stoichiometry, temporality, and the cumulative effects of all interactions within the specific biological system. Additionally, functions that deviate from the dogmatic interaction model of microRNA as negative regulator of protein targets expression, such as the micropeptide coding potential of precursors or direct binding of Toll-like receptors (25), represent only a fraction of their described biological versatility. We clearly know that microRNAs provide valuable insights into cellular status and disease-associated genomic alterations. Therefore, harnessing this potential for early diagnosis and risk assessment by integrating microRNAs into disease-specific transcriptional profiles may unlock their full therapeutic promise. It will be the creativity and the perseverance of next generation of RNA biologists and clinician scientists who will pave the way to the successful clinical applications for microRNAs.

## Lessons from the scientific endeavors of Victor Ambros and Gary Ruvkun

The Nobel Prize has recognized the importance of microRNAs, marking the beginning of a new renaissance for the field. The award highlights the fundamental role of microRNAs in physiology and evolution and the outmost importance of the basic research and fundamental biology discoveries from model organisms. Although modern medicine’s research outcomes are clinical applications for patients, it is the basic scientist’s curiosity and hypothesis-driven research that is pushed forward the scientific endeavor. Advances in synthetic biology, computational modeling, big-data analyses, and artificial intelligence methods offer promising tools to address the RNA research challenges, including the therapeutic developments.

The discovery of microRNAs was a groundbreaking milestone that not only advanced scientific knowledge but also provided enduring lessons for researchers at any stage of their career. Key among these is the importance of strong mentorship, as Victor Ambros and Gary Ruvkun trained under Nobel laureates Robert Horvitz and Walter Gilbert, whose labs fostered innovation and collaboration and nurtured scientific brilliance. Their work also exemplified perseverance, as they overcame technical limitations in *C*. *elegans* cloning methods and drove publishing policy changes, such as Ambros persuading *Cell* to adopt multiple-first-author guidelines (5). Collaboration further played a critical role, with their exchange of sequence data linking lin-4 and lin-14, showcasing the power of collegial trust in advancing science. Their ambition to stay competitive was evident in the relentless effort by Ambros and Rosalind Lee to finalize their 2001 *Science* manuscript in time to be published alongside work by Bartel and Tuschl’s groups. As beneficiaries of their discoveries, we can echo Ambros’s tribute to Rosalind Lee (26), “Without you, nothing — with you, everything,” by extending it to say that without Ambros, Ruvkun, and Lee, microRNAs would not exist; with their contributions, the field of microRNA research was brought to life.

## Version 1 12/10/2024

In-Press Preview

## Version 2 01/16/2025

Electronic publication

## Version 3 02/03/2025

Corrected reference 8
